# Influence of Maternal Fish Oil Supplementation on the Risk of Asthma or Wheeze in Children: A Meta-Analysis of Randomized Controlled Trials

**DOI:** 10.3389/fped.2022.817110

**Published:** 2022-02-21

**Authors:** Shaojing Wu, Changhong Li

**Affiliations:** ^1^Department of Clinical Nutrition, Hainan Maternal and Children's Medical Center, Changbin Road Children's Hospital, Haikou, China; ^2^Department of Obstetrics, Hainan Maternal and Children's Medical Center, Changbin Road Children's Hospital, Haikou, China

**Keywords:** fish oil, asthma, prenatal, infancy, meta-analysis

## Abstract

**Background:**

Previous studies evaluating the influences of maternal fish oil supplementation on the risk of asthma or wheeze in children showed inconsistent results. We performed a meta-analysis or randomized controlled trials (RCTs) to systematically evaluate the efficacy of maternal fish oil supplementation for asthma or wheeze.

**Methods:**

Relevant RCTs were obtained by search of PubMed, Embase, and Cochrane's Library databases. A random-effects model incorporating the potential publication bias was used to pool the results.

**Results:**

Ten RCTs with 3,676 infants were included. Compared to control, maternal supplementation with fish oil was not associated with a reduced risk of asthma or wheeze [odds ratio (OR): 0.91, 95% confidence interval (CI): 0.72–1.14, *P* = 0.40] with mild heterogeneity (*I*^2^ = 28%). Subgroup analyses showed that maternal fish oil supplementation significantly reduced the risk of asthma (OR: 0.56, 95% CI: 0.35–0.91, *P* = 0.02; *I*^2^ = 0%), but not the risk of wheeze (OR: 1.12, 95% CI: 0.90–1.41, *P* = 0.32; *I*^2^ = 0%). In addition, maternal fish oil supplementation was associated with reduced risk of asthma or wheeze in high-dose studies (≥1,200 mg/d, OR: 0.65, 95% CI: 0.48–0.87, *P* = 0.003; *I*^2^ = 0%), but not in low-dose studies (<1,200 mg/d, OR: 1.10, 95% CI: 0.88–1.38, *P* = 0.39; *I*^2^ = 0%, *P* for subgroup difference = 0.005). Study characteristics such as the risk of the infants, timing of supplementation, and follow-up duration did not significantly affect the results.

**Conclusions:**

Maternal fish oil supplementation may reduce the risk of clinically diagnosed asthma in children, particularly with high-dose fish oil.

## Introduction

Asthma and wheeze (asthma/wheeze) is a respiratory syndrome which mainly occurs in early childhood (1–3). The pathogenesis of asthma/wheeze is complicated, which involves a variety of inflammatory cells and cytokines, leading to chronic airway inflammation, airway hypersensitivity, and bronchial airflow limitation (4, 5). According to epidemiological studies, the incidence of asthma/wheeze is increasing in recent decades, which has become an important threat to the health of the global population, particularly for the children and adolescents (1, 2). Therefore, identification of effective preventative strategy for asthma/wheeze is of great clinical significance (6). Previous epidemiological studies have suggested that prenatal maternal or postnatal infancy fish oil supplementation may be associated with lower risk of allergic diseases in early childhood, including asthma (7, 8). Accordingly, maternal supplementation of fish oil, which mainly consists of the marine omega-3 polyunsaturated fatty acids (n-3 PUFAs) eicosapentaenoic acid (EPA) and docosahexaenoic acid (DHA), has been expected to reduce the incidence of asthma/wheeze (9). However, previous randomized controlled trials (RCTs) evaluating the influences of maternal fish oil supplementation on the risk of asthma or wheeze in children showed inconsistent results (10–19). Although some RCTs supported that prenatal maternal supplementation of fish oil reduced the risk of asthma/wheeze in offspring (16, 17), the others did not (10–15, 18, 19). Therefore, we performed a meta-analysis of RCTs to systematically evaluate the efficacy of maternal fish oil supplementation for asthma/wheeze. Comprehensive subgroup analyses were also performed to explore the potential influences of study characteristics on the outcome.

## Methods

We followed the instructions of the PRISMA (Preferred Reporting Items for Systematic Reviews and Meta-Analyses) statement (20) and the Cochrane Handbook guidelines (21) during the designing, performing, and reporting of the meta-analysis.

### Search Strategy

PubMed, Embase, and the Cochrane Library (Cochrane Center Register of Controlled Trials) databases were searched for relevant studies with a combined strategy of: (1) “omega-3 fatty acids” OR “fish oil” OR fish-oil OR “polyunsaturated fatty acids” OR “marine oil” OR “eicosapentaenoic acid” OR “docosahexaenoic acid” OR “DHA” OR “EPA”; (2) “asthma” OR “wheeze” OR “wheezing” OR “pulmonary” OR “lung” OR “allergy” OR “allergic”; (3) “child” OR “children” OR “adolescent” OR “pediatric” OR “pediatric” OR “infant” OR “neonate” OR “newborn” OR “toddler”; and (4) “random” OR “randomly” OR “randomized” OR “randomized”. Only clinical studies were considered. The references of related reviews and original articles were also searched as a complementation. The latest database search was conducted on April 5th, 2021.

### Study Selection

Inclusion criteria were: (1) peer-reviewed articles in English; (2) designed as parallel-group RCTs; (3) included infants who were randomly allocated to an intervention group of maternal fish oil supplementation or a control group of placebo or blank treatment; prenatal supplementation of fish oil was achieved by maternal intake during gestational periods and postnatal supplementation was achieved by maternal intake during breast feeding; and (4) reported the incidence of asthma and/or the symptom of wheeze of the offspring during follow-up. If studies with overlapped population were retrieved, the one with the longest follow-up duration was included. Diagnosis and definition of asthma/wheeze were in accordance with those applied among the original studies. Reviews, preclinical studies, observational studies, crossover RCTs, studies with overlapped population, and studies that did not report related outcomes were excluded.

### Data Extraction and Quality Assessment

Study search, data extraction, and quality evaluation were achieved by two independent authors. If disagreement occurred, it was resolved by consensus between the two authors. We extracted data regarding study information (first author, publication year, and study country), study design (blind or open-label), maternal or birth information, intervention of fish oil supplementation (dosage, timing and durations), regimen of controls, number of children followed, and outcomes reported. Quality evaluation was achieved using the Cochrane's Risk of Bias Tool (21) according to the following aspects: (1) random sequence generation; (2) allocation concealment; (3) blinding of participants and personnel; (4) blinding of outcome assessors; (5) incomplete outcome data; (6) selective outcome reporting; and (7) other potential bias. A total score of 5–7, 3–4, and 0–2 indicated high, moderate, and low quality of the included study.

### Statistical Analysis

Incidence of asthma in each arm was evaluated *via* odds ratio (OR) and its 95% confidence intervals (CIs). We used the Cochrane's Q test to detect the heterogeneity, and significant heterogeneity was suggested if *P* < 0.10 (22). The *I*^2^ statistic was also calculated, and an *I*^2^ > 50% reflected significant heterogeneity. Pooled analyses were calculated using a random-effect model because this method incorporates the influence of potential heterogeneity and retrieves a more generalized result (21). Sensitivity analyses by excluding one dataset at a time were used to evaluate the stability of the findings. Subgroup analyses comparing the results according to the differences of outcomes reported, infant characteristics (normal or high-risk of asthma), dose of fish oil, timing of intervention, and follow-up durations were performed. For continuous variables, medians were used for cut-off. Publication bias was evaluated by visual inspection of funnel plots, and the Egger's regression asymmetry test (23). *P* < 0.05 were considered statistically significant. The RevMan (Version 5.1; Cochrane, Oxford, UK) and Stata software (Version 12.0; Stata, College Station, TX, USA) were applied for statistical analyses.

## Results

### Search Results

**Figure 1:**
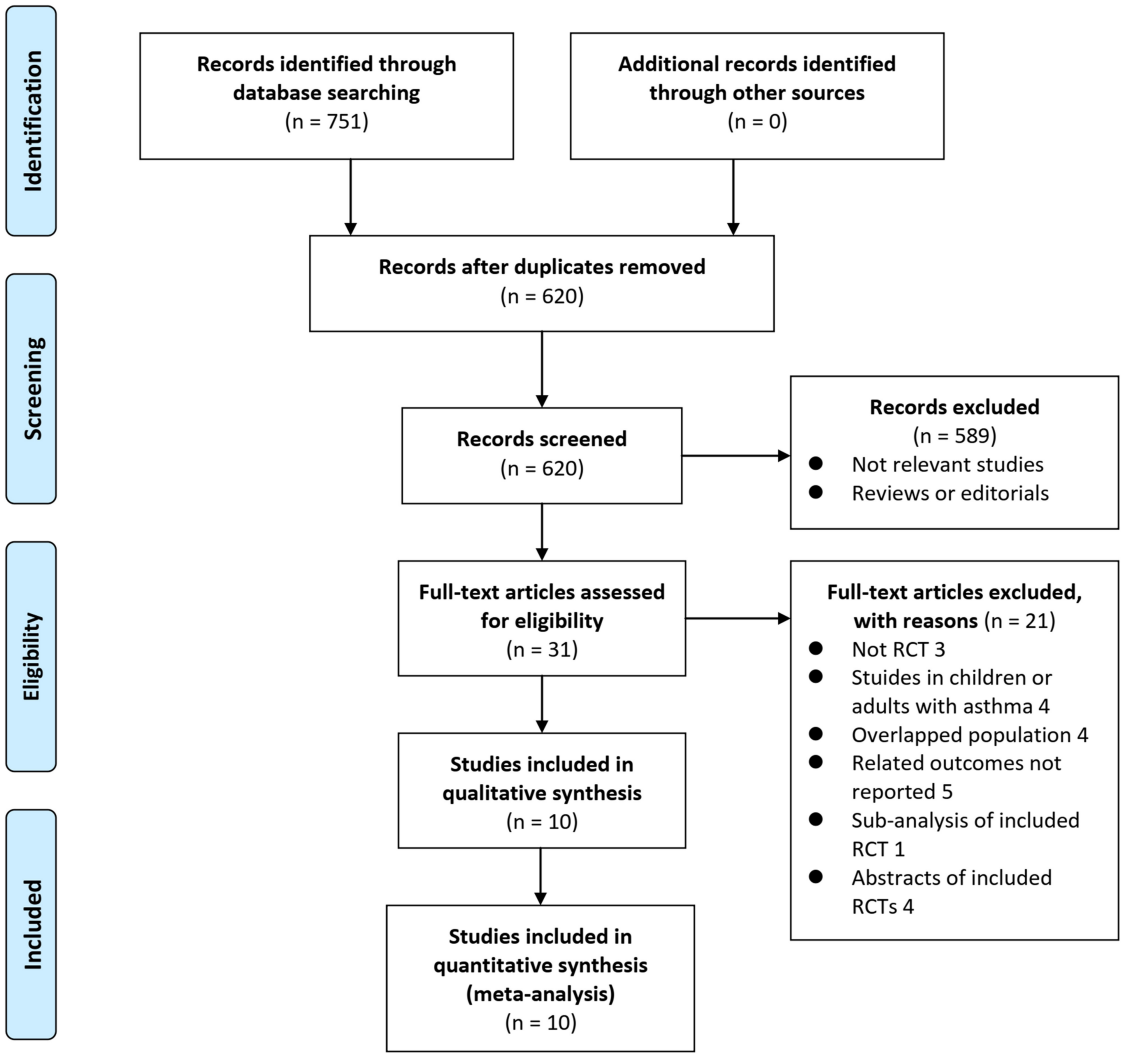
Flowchart of literature search. From Moher et al. (24).

In summary, 751 articles were obtained through the database search. After exclusion of duplicate studies, 620 articles were screened. Among them, 589 articles were subsequently excluded based on titles and abstracts primarily because these studies were irrelevant. Among the 31 potentially relevant articles, 21 were further excluded *via* full-text review based on reasons listed in Figure 1. Finally, 10 RCTs (10–19) were included in the meta-analysis.

### Study Characteristics

Table 1 shows the characteristics of the included studies. Overall, 10 RCTs were including a total of 3,676 infants were included (10–19). Since one study reported two interventional arms with different doses of fish oil (15), these datasets were included in the meta-analysis separately. These studies were performed in Australia (10, 13, 18, 19), Sweden (11), Denmark (16, 17), UK (14), USA (15), and Mexico (12), respectively. The supplementation of fish oil was achieved by prenatally maternal intake during gestational periods in seven studies (10, 12, 14–18), by postnatally maternal intake during breast feeding in two studies (13, 19), and by pre- and post-natal intake in another study (11). The dose of n-3 PUFAs varied from 390 to 3,700 mg/d, and the doses of EPA and DHA ranged within 0~1,600 and 270~2,240 mg/d. The follow-up durations varied from 6 months to 24 years. Outcome of clinically diagnosed asthma was reported in three studies (10, 11, 17), symptoms of wheeze in five studies (12–14, 18, 19), and outcome of asthma or wheeze in two studies (15, 16). For the studies reporting the outcomes of clinically diagnosed asthma, two studies defined the outcome as “doctor diagnosed wheezing at least three times during the first 2 years based on the medical records” (10, 11), and another study used asthma medication prescription records and/or asthma discharge diagnosis as the validation of the outcome (17).

**Table 1:** Characteristics of the included RCTs.

**References**	**Country**	**Study design**	**Maternal/birth characteristics**	**n-3 PUFAs** **dose**	**EPA** **dose**	**DHA** **dose**	**Control**	**Intervention duration**	**Follow-up** **duration**	**No. of children followed**	**Outcome reported**
				**mg/d**	**mg/d**	**mg/d**			**Years**		
Dunstan et al. (10)	Australia	R, DB, PC	Atopic pregnant women	3,700	1,460	2,240	Olive oil	Prenatal: from 20 week GA to delivery	1	83	Clinically diagnosed asthma
Furuhjelm et al. (11)	Sweden	R, DB, PC	Women at risk of having allergic infant	2,700	1,600	1,100	Soya bean oil	Prenatal and postnatal: from 25 week GA to 3.5 months of breast feeding	2	119	Clinically diagnosed asthma
Imhoff-Kunsch et al. (12)	Mexico	R, DB, PC	Women with normal pregnancy	400	0	400	Soy oil	Prenatal: from 18~22 week GA to delivery	0.5	834	Wheeze symptom
D'Vaz et al. (13)	Australia	R, DB, PC	Women at risk of having allergic infant	390	110	280	Olive oil	Postnatal: from delivery to 6 months of breast feeding	1	241	Wheeze symptom
Noakes et al. (14)	UK	R, SB	Women with normal pregnancy	495	165	330	Regular diet	Prenatal: from 20 week GA to delivery	0.5	83	Wheeze symptom
Berman et al. (15)	USA	R, DB, PC	Pregnant women with history of depression	1,334	1,060	274	Soy oil	Prenatal: from 12 week GA to delivery	3	44	Asthma or wheezing
Berman et al. (15)	USA	R, DB, PC	Pregnant women with history of depression	1,080	180	900	Soy oil	Prenatal: from 12 week GA to delivery	3	40	Asthma or wheezing
Bisgaard et al. (16)	Denmark	R, DB, PC	Population based women with normal pregnancy	2,400	1,440	960	Olive oil	Prenatal: from 24 week GA to delivery	4	695	Persistent wheeze or asthma
Hansen et al. (17)	Denmark	R, SB, PC	Population based women with normal pregnancy	2,700	1,570	1,130	Olive oil	Prenatal: from 30 week GA to delivery	24	402	Clinically diagnosed asthma
Best et al. (18)	Australia	R, DB, PC	Women at risk of having allergic infant	900	100	800	Vegetable oil	Prenatal: from 21 week GA to delivery	6	566	Wheeze symptom
Gunaratne et al. (19)	Australia	R, DB, PC	Women with Infants born at <33 week gestation	500	0	500	Soy oil	Postnatal: from delivery to 2 months of breast feeding	7	569	Wheeze symptom

*RCT, randomized controlled trials; R, randomized; DB, double-blinded; PC, placebo-controlled; SB, single-blinded; EPA, eicosapentaenoic acid; DHA, docosahexaenoic acid; GA, gestational age; n-3 PUFAs, omega-3 polyunsaturated fatty acids*.

### Data Quality

Table 2 shows the details of study quality evaluation. Eight of the included studies was double-blind (10–16, 18, 19), while the other two was single-blind (14, 17). Methods of random sequence generation were reported in seven studies (12–16, 18, 19), and information of allocation concealment were reported in three studies (10, 16, 18). The overall quality score varied between 4 and 7, which suggested generally moderate to good study quality.

**Table 2:** Details of study quality evaluation *via* the Cochrane's Risk of Bias tool.

**References**	**Random sequence generation**	**Allocation concealment**	**Blinding of participants**	**Blinding of outcome assessment**	**Incomplete outcome data addressed**	**Selective reporting**	**Other sources of bias**	**Total**
Dunstan et al. (10)	Unclear	Yes	Yes	Yes	Yes	Yes	Yes	6
Furuhjelm et al. (11)	Unclear	Unclear	Yes	Yes	Yes	Yes	Yes	5
Imhoff-Kunsch et al. (12)	Yes	Unclear	Yes	Yes	Yes	Yes	Yes	6
D'Vaz et al. (13)	Yes	Unclear	Yes	Yes	Yes	Yes	Yes	6
Noakes et al. (14)	Unclear	Unclear	Unclear	Yes	Yes	Yes	Yes	4
Berman et al. (15)	Yes	Unclear	Yes	Yes	Yes	Yes	Yes	6
Berman et al. (15)	Yes	Unclear	Yes	Yes	Yes	Yes	Yes	6
Bisgaard et al. (16)	Yes	Yes	Yes	Yes	Yes	Yes	Yes	7
Hansen et al. (17)	Unclear	Unclear	Yes	Unclear	Yes	Yes	Yes	4
Best et al. (18)	Yes	Yes	Yes	Yes	Yes	Yes	Yes	7
Gunaratne et al. (19)	Yes	Unclear	Yes	Yes	Yes	Yes	Yes	6

### Meta-Analysis Results

**Figure 2:**
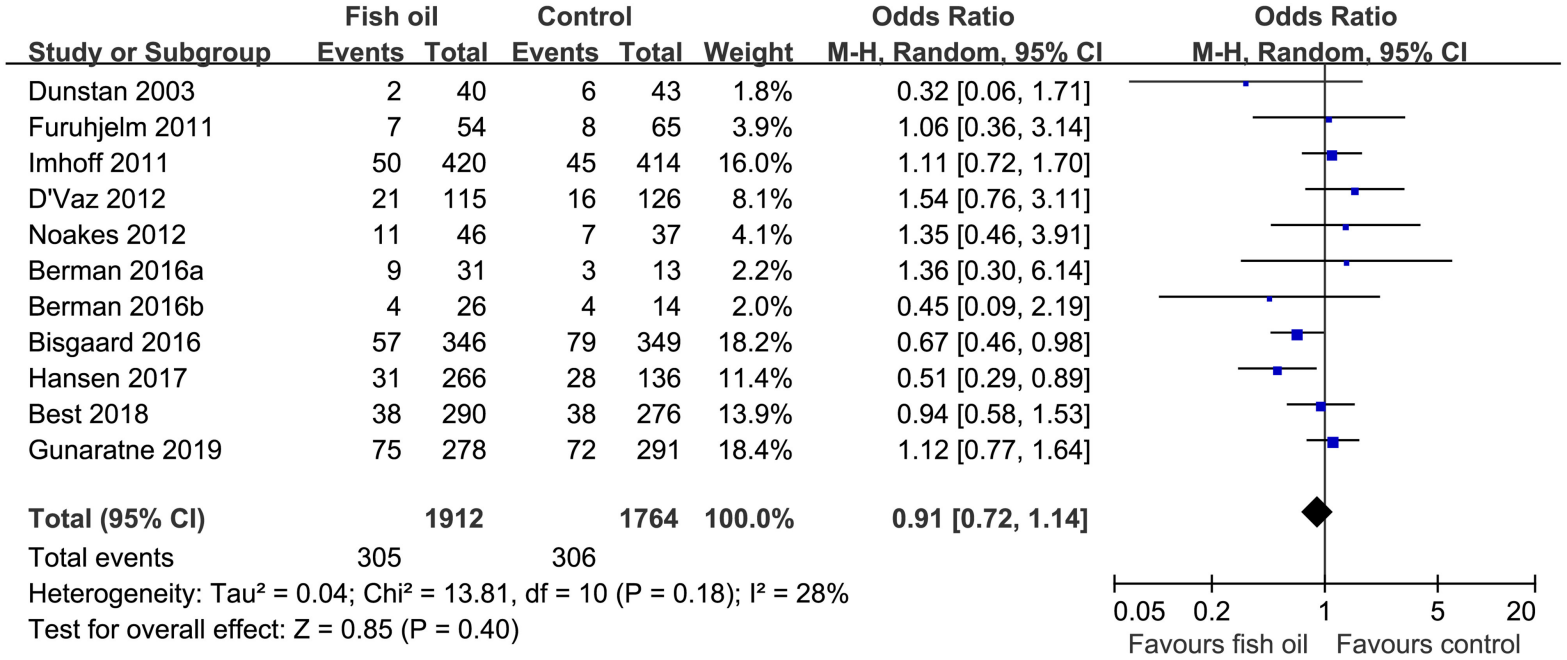
Forest plots for the meta-analysis of the influence of maternal fish oil supplementation on the risk of asthma or wheeze during follow-up.

Pooled results of 11 datasets including 3,676 infants showed that compared to control, maternal supplementation with fish oil was not associated with an overall reduced risk of asthma or wheeze (OR: 0.91, 95% CI: 0.72–1.14, *P* = 0.40; Figure 2) with mild heterogeneity (*I*^2^ = 28%). Further sensitivity analysis by excluding one dataset at a time showed consistent results (OR: 0.85~0.98, *P* all > 0.05). Subgroup analyses showed that maternal fish oil supplementation may reduce the risk of clinically diagnosed asthma (OR: 0.56. 95% CI: 0.35–0.91, *P* = 0.02), but not the risk of wheeze (OR: 1.12, 95% CI: 0.90–1.41, *P* = 0.32; Table 3). In addition, maternal fish oil supplementation was associated with reduced risk of asthma or wheeze in high-dose studies (≥1,200 mg/d), but not in low-dose studies (<1,200 mg/d, P for subgroup difference = 0.005; Table 3). Study characteristics such as the risk of the infants, timing of supplementation, and follow-up duration did not significantly affect the results (*P* for subgroup difference all > 0.05; Table 3).

**Table 3:** Subgroup analyses.

	**Risk of asthma or wheeze symptom**				
**Characteristics**	**No. of datasets (infants)**	**OR (95% CI)**	***I* ^2^**	***P* for subgroup effect**	***P* for subgroup difference**
**Outcomes**					
Asthma	3 (604)	0.56 [0.35, 0.91]	0%	0.02	
Wheeze	5 (2,293)	1.12 [0.90, 1.41]	0%	0.32	
Asthma or wheeze	3 (779)	0.69 [0.48, 0.98]	0%	0.04	0.009
**Infant characteristics**					
High-risk	4 (1,009)	1.04 [0.70, 1.53]	7%	0.86	
Normal	7 (2,667)	0.86 [0.64, 1.15]	40%	0.31	0.45
Dose of fish oil					
<1,200 mg/d	6 (2,333)	1.10 [0.88, 1.38]	0%	0.39	
≥1,200 mg/d	5 (1,343)	0.65 [0.48, 0.87]	0%	0.003	0.005
**Dose of EPA**					
<1,000 mg/d	6 (2,333)	1.10 [0.88, 1.38]	0%	0.39	
≥1,000 mg/d	5 (1,343)	0.65 [0.48, 0.87]	0%	0.003	0.005
**Dose of DHA**					
<800 mg/d	5 (1,771)	1.18 [0.92, 1.52]	0%	0.19	
≥800 mg/d	6 (1,905)	0.70 [0.54, 0.89]	0%	0.004	0.003
**Timing of intervention**					
Pre-natal	8 (2,747)	0.80 [0.61, 1.04]	22%	0.09	
Post-natal	2 (810)	1.20 [0.86, 1.68]	0%	0.27	
Pre- and post-natal	1 (119)	1.06 [0.36, 3.14]	–	0.91	0.16
**Follow-up duration**					
<3 years	5 (1,360)	1.15 [0.83, 1.59]	0%	0.40	
≥3 years	6 (2,316)	0.80 [0.60, 1.07]	36%	0.14	0.11

*OR, odds ratio; CI, confidence interval; EPA, eicosapentaenoic acid; DHA, docosahexaenoic acid*.

### Publication Bias

**Figure 3:**
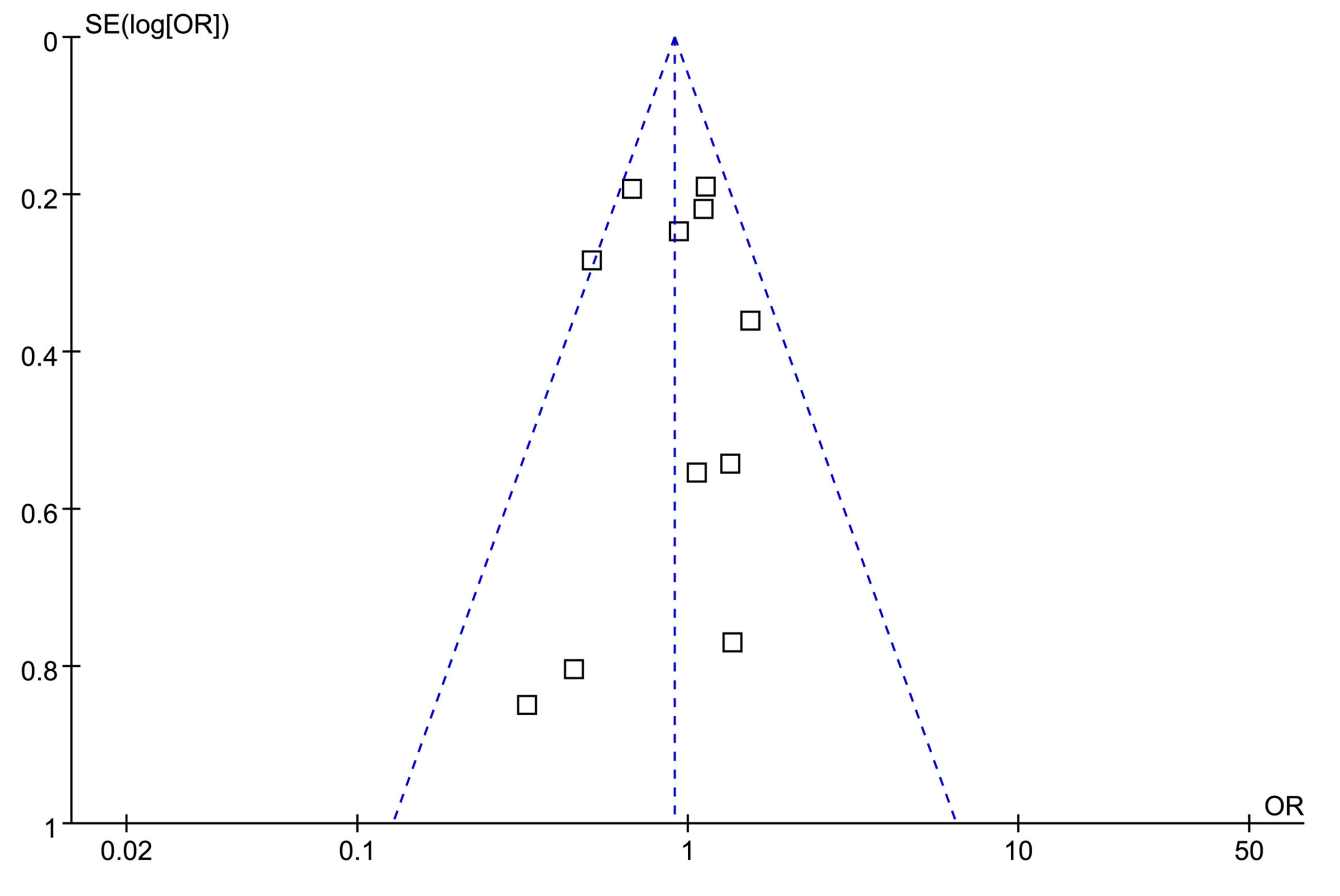
Funnel plots for the publication bias of the meta-analysis.

The funnel plots were symmetrical for the overall meta-analysis, suggesting low risk of publication bias (Figure 3). Egger's regression tests also showed low risk of publication bias (*P* for Egger's regression test = 0.652).

## Discussion

In this meta-analysis of RCTs, we found that maternal fish oil supplementation was not associated with an overall reduced risk of asthma/wheeze in children. However, subgroup analysis suggested that maternal fish oil supplementation may reduce the risk of clinically diagnosed asthma, but not for the overall wheeze symptoms. Besides, maternal fish oil supplementation reduced the risk of asthma/wheeze in studies with high-dose fish oil (≥1,200 mg/d), but not in those with low-dose fish oil (<1,200 mg/d). Characteristics of infants (high risk of allergic disease or normal) and timing of supplementation (prenatal or postnatal) did not seem to significantly affect the results. Taken together, results of the meta-analysis indicated that maternal fish oil supplementation may reduce the risk of clinically diagnosed asthma in children, particularly with high-dose fish oil.

Several systematic reviews and meta-analyses have been published previously to evaluate the association between maternal fish oil supplementation and risk of allergic diseases, including asthma and wheeze. Early meta-analyses mainly included observational studies, which suggested that fish or fish oil intake may be beneficial to prevent asthma in children (7, 8). However, the inherited methodological of observational studies, such as the recall and selection biases and confounding effects prevented these studies to drawl a confirmed conclusion (7, 8). Subsequently, a pooled analysis including 18 European and USA birth cohorts published before 2017 did not support a potential association of fish and seafood consumption during pregnancy with the risk of asthma/wheeze in offspring (25). Besides, a meta-analysis including one RCT and 13 prospective cohort studies showed that fish intake in infancy could reduce the risk of eczema and allergic rhinitis in children, whereas maternal fish intake during pregnancy does not affect any atopic outcome, including asthma/wheeze (26). Similarly, these findings were also based on the results of observational studies, which should be interpreted cautiously. Recently, two meta-analyses have been published to evaluate the influence of prenatal fish oil supplementation during pregnancy on allergic diseases, including asthma/wheeze in offspring (27, 28). One of the meta-analysis included seven RCTs published before 2017 showed that prenatal fish oil supplementation during pregnancy did not significantly reduce the risk of asthma/wheeze in offspring (27). However, due to the limited datasets included, the authors failed to evaluate the outcome of asthma and wheeze separately. Besides, no subgroup analyses were performed to evaluate the possible influences of key study characteristics on the outcome, such as the risk stratification of the infants and dose of fish oil (27). The other meta-analysis, by including seven RCTs published before 2018, showed that supplementation during pregnancy may reduce the incidence of wheeze/asthma of children (28). However, two studies (one original and one extension) of the same population were both included into the meta-analysis (29, 30), which seriously confounded the results of the meta-analysis.

Compared to the previous meta-analyses, our study has multiple methodological strengths. Firstly, by including the most up-to-date studies, our meta-analysis is comprised of the largest datasets and sample size (11 datasets including 3,676 infants) which could provide an updated view regarding the role of maternal fish oil supplementation on the risk of asthma/wheeze in childhood. As an update, a study of the same population as the previous two studies (29, 30) but with longest follow-up duration has been included in our study (18). Secondly, sensitivity analyses by excluding one study at a time showed that the results of the meta-analysis was not primarily driven by either of the included studies, indicating the stability of the finding. Finally, multiple predefined subgroup analysis was performed to evaluate the potential influence of study characteristics on the outcome, which may retrieve some interesting findings. Results of our meta-analysis showed that maternal fish oil supplementation may reduce the risk of clinically diagnosed asthma, but not the wheeze symptom in children. It has been indicated that not all children with wheeze will be eventually diagnosed as asthma (31, 32). Unlike asthma, preschool wheeze may have multiple causes other than those also involved in the pathogenesis of asthma (33). Therefore, preventative strategies for asthma may not be adequate for the prevention of wheeze. Future clinical trials are recommended to separately report the potential influence of fish oil supplementation on wheeze and asthma. Besides, results of subgroup analyses also showed that maternal fish oil supplementation was associated with reduced risk of asthma or wheeze in high-dose studies (≥1,200 mg/d), but not in low-dose studies (<1,200 mg/d). These findings are consistent with the previous observations which showed dose-dependent anti-inflammatory (34, 35) and immune modulatory (36–38) effects of fish oil. In this regard, a relative high-dose of fish oil is recommended for future clinical trials to evaluate the role of maternal fish oil supplementation on asthma risk in children.

Our study also has limitations. Firstly, according to the Global Initiative for Asthma Strategy 2021, it may be challenging to make a confident diagnosis of asthma under 5 years old (39). For the three included RCTs that reported the outcome of clinically diagnosed asthma (10, 11, 17), two of them had follow-up duration of <5 years and the diagnosis of asthma in these studies may need to be further validated. Therefore, results of subgroup analysis that maternal fish oil supplementation may reduce the risk of clinically diagnosed asthma should be interpreted with caution, and more RCTs with longer follow-up duration and evidence-based diagnosis of asthma should be performed to validate these findings. In addition, maternal and infantile dietary factors were generally not controlled among the included studies, which may affect the outcome of the meta-analysis. In addition, some other maternal-infantile nutritional factors may also affect the potential influences of fish oil supplementation on asthma/wheeze, such as concurrent use of some other nutritional supplements, which should also be analyzed in future studies (40). Besides, the optimal dose, timing, and regimens for fish oil supplementation remains to be determined to maximize its potential preventative efficacy on clinically diagnosed asthma in children. Future studies are still warranted. Finally, although high-dose fish oil is recommended based on the results of subgroup analysis, the safety of maternal high-dose fish oil supplementation should be evaluated in the future.

In conclusion, results of this updated meta-analysis showed that maternal fish oil supplementation may reduce the risk of clinically diagnosed asthma in children, particularly with high-dose fish oil. These findings should be validated in future clinical trials, and the safety of maternal high-dose fish oil supplementation should also be assessed.

## Data Availability Statement

The original contributions presented in the study are included in the article/supplementary material, further inquiries can be directed to the corresponding author.

## Author Contributions

SW and CL designed the study, performed literature search, data extract, and statistical analyses. SW drafted the manuscript. CL revised the manuscript. All authors approved the submission. All authors contributed to the article and approved the submitted version.

## Funding

This study was supported by Hainan Provincial Natural Science Foundation of China (821QN1002) and Hainan Province Clinical Medical Center.

## Conflict of Interest

The authors declare that the research was conducted in the absence of any commercial or financial relationships that could be construed as a potential conflict of interest.

## Publisher's Note

All claims expressed in this article are solely those of the authors and do not necessarily represent those of their affiliated organizations, or those of the publisher, the editors and the reviewers. Any product that may be evaluated in this article, or claim that may be made by its manufacturer, is not guaranteed or endorsed by the publisher.
